# At the Leading Front of Neuroscience: A Bibliometric Study of the 100 Most-Cited Articles

**DOI:** 10.3389/fnhum.2017.00363

**Published:** 2017-07-21

**Authors:** Andy W. K. Yeung, Tazuko K. Goto, W. Keung Leung

**Affiliations:** ^1^Oral and Maxillofacial Radiology, Applied Oral Sciences, Faculty of Dentistry, University of Hong Kong Hong Kong, Hong Kong; ^2^Department of Oral and Maxillofacial Radiology, Tokyo Dental College Tokyo, Japan; ^3^Periodontology, Faculty of Dentistry, University of Hong Kong Hong Kong, Hong Kong

**Keywords:** bibliometrics, functional neuroimaging, information science, literature-based discovery, neurosciences, publications

## Abstract

**Background:** It might be difficult for clinicians and scientists to identify comprehensively the major research topics given the large number of publications. A bibliometric report that identifies the most-cited articles within the body of the relevant literature may provide insight and guidance for readers toward scientific topics that are considered important for researchers and all relevant workers of academia. To our knowledge, there is a lack of an overall evaluation of the most-cited articles and hence of a comprehensive review of major research topics in neuroscience. The present study was therefore proposed to analyze and characterize the 100 most-cited articles in neuroscience.

**Methods:** Based on data provided from Web of Science, the 100 most-cited articles relevant to neuroscience were identified and characterized. Information was extracted for each included article to assess for the publication year, journal published, impact factor, adjusted impact factor, citation count (total, normalized, and adjusted), reference list, authorship and article type.

**Results:** The total citation count for the 100 most-cited articles ranged from 7,326 to 2,138 (mean 3087.0) and the normalized citation count ranged from 0.163 to 0.007 (mean 0.054). The majority of the 100 articles were research articles (67%) and published from 1996 to 2000 (30%). The author and journal with the largest share of these 100 articles were Stephen M. Smith (*n* = 6) and *Science* (*n* = 13) respectively. Among the 100 most-cited articles, 37 were interlinked via citations of one another, and they could be classified into five major topics, four of which were scientific topics, namely neurological disorders, prefrontal cortex/emotion/reward, brain network, and brain mapping. The remaining topic was methodology. Interestingly 41 out of 63 of the rest, non-interlinked articles could also be categorized under the above five topics. Adjusted journal impact factor among these 100 articles did not appear to be associated with the corresponding adjusted citation count.

**Conclusion:** The current study compiles a comprehensive list and analysis of the 100 most-cited articles relevant to neuroscience that enables the comprehensive identification and recognition of the most important and relevant research topics concerned.

## Introduction

Neuroscience is a broad research field aimed at investigating the relationships among neural structures, functions and behaviors. It comprises many research themes; examples include analytic methods, the acquisition of functional neuroimaging and its physics, computational modeling and analytics, physiology and anatomy, sensorimotor functions, aging and cognitive functions, social neuroscience, and language and learning. With the enormous number of articles published in the field, it might be daunting for clinicians and scientists to identify the major research topics of the field concerned. A bibliometric report that identifies the research hotspots and their contributors may provide updated insight as a first step to further explore the details (Snyder and Raichle, 2012; Kim, H. J. et al., 2016; Yeung et al., 2017a,b).

When citation analysis is properly used, it could objectively evaluate the scientific performances of a selected corpus of literature at a relatively low financial cost (Garfield, 1979). There are several up-to-date publications that report on the 100 most-cited articles in various subfields of neuroscience, such as neurointervention (Kim, E. S. et al., 2016), neuroimaging (Kim, H. J. et al., 2016), neurorehabilitation (Kreutzer et al., 2017) and neurosurgery (Ponce and Lozano, 2010). However, to our knowledge, there is a lack of published papers that provide an overall evaluation of the most-cited neuroscience articles.

The purpose of the present study was therefore to identify the 100 most-cited articles in neuroscience and to analyze their characteristics and inter-citation network.

## Materials and methods

### Data source

The choice of data source and the search strategy were adopted from previous reports (Ponce and Lozano, 2010; Kim, E. S. et al., 2016; Kim, H. J. et al., 2016; Kreutzer et al., 2017). The source of data was the Clarivate Analytics-owned, Web-based, multidisciplinary database Web of Science (WoS), which provides the bibliometric data of published scientific articles starting from 1945. In March 2017, we searched in the Science Citation Index Expanded (SCI-E) section of WoS to identify articles with the following string: TS = neuro^*^ OR WC = Clinical Neurology OR WC = Medicine, General & Internal OR WC = Neuroimaging OR WC = Neuroscience OR WC = Radiology, Nuclear medicine & Medical imaging. This string searched for articles that: (1) contain the string “neuro” in the title, abstract, or keywords; or (2) are published in journals that belong to the five selected Journal Citation Report (JCR) categories.

The articles were sorted according to citation count, and their titles and abstracts were evaluated to ensure that they were relevant to neuroscience. No restrictions were placed on the type of research model (*in vivo / in vitro*), article type (e.g., research article, review, editorial, letter, etc.), or publication language. The articles citing the 100 most-cited articles and the reference lists of the 100 most-cited articles were searched to decrease the chance that relevant neuroscience papers with equal or higher citations were missed.

### Data extraction

The 100 most-cited articles were reviewed and the following information extracted: (1) year of publication; (2) title of journal; (3) adjusted journal impact factor (i.e., impact factor per year since publication of that article); (4) total citation count; (5) normalized citation count (i.e., citation count received during the first 10 years after publication divided by neuroscience publication count during that period); (6) adjusted citation count (i.e., citation count per year since publication of that article); (7) reference list; (8) authorship; and (9) article type. Bradford's law of scattering was applied to the data of the 100 most-cited articles to investigate if core articles are responsible for one-third of the total citations that these articles received (Vickery, 1948). Based on Bradford's law, three groups of articles should contribute to the total citation count when it is equally divided into three portions, whereas the number of articles within the groups should be in the ratio of 1:n:n^2^. In addition, the Highly Cited Researchers 2016 list was searched to identify if any authors of these 100 most-cited articles have “contributed markedly high numbers of top-cited papers over a recent eleven year period, 2004–2014”^1^ In addition, we identified if any authors of these 100 articles received any major scientific awards (Lasker Award^2^ or Nobel Prize^3^.

### Citation network

To reveal if these 100 articles were interlinked (i.e., citing one another), a citation network consisting of these interlinked articles was visualized by using VOSviewer (Van Eck and Waltman, 2009). Each article was represented by a bubble. Two bubbles were connected by a line if either one of them cited the other. In this visualized network, the bubbles were categorized manually according to their topics. Each topic was defined as a discrete theme serving as an umbrella term, under which papers concerning related research work could be grouped together. Bubble size indicated the citation count received within the network.

### Influencing factors of citation count

Because the article citation count influences the journal impact factor, and because a higher impact factor tends to attract academics' attention, we assumed there might be a possibility that a higher adjusted impact factor would lead to a higher adjusted citation count. Here, we calculated adjusted values instead of normalized values because online WoS database and the authors' institutions do not have impact factor data before 1997. Therefore, to investigate the association involving impact factor, it was more meaningful to use adjusted data instead of normalizing them according to statistics from a 10-year period. Pearson's correlation test was applied.

Next, we applied Pearson's correlation test to investigate the association between normalized citation count and years since publication. In the old times there were fewer publications so it was reasonable to expect that the older publications received higher normalized citation count.

Statistical tests were performed with SPSS (Version 23.0, IBM, NY, US), and the test results were significant if *p* < 0.05. If the test results were insignificant but suggested data clustering in the scatter plot, we would conduct a two-step cluster analysis to evaluate if the involved citation count (adjusted or normalized) could be separated into clusters. Significant results would be further examined with Price's law by applying the best-fitting linear or exponential trend lines to the scatter plot. If the data had a better fit by the exponential line, results would be considered to fulfill Price's law, which predicts an exponential change of an outcome variable (i.e., adjusted or normalized citation count in the current study) over the survey period (López-Muñoz et al., 2014; Yeung et al., 2017b). For illustrative purposes, the data would be plotted against the year of publication (instead of years since publication) together with annual counts of neuroscience publications to examine if both trends followed an exponential growth consistent with the notion of “publish or perish.” The annual counts of neuroscience publications were acquired by searching “neuro^*^” in the PubMed database.

## Results

The 100 most-cited articles were sorted in Table 1 according to normalized (Rank A) and total (Rank B) citation counts. The total number of citations that the 100 most-cited articles in the neuroscience field have received ranged from 2,138 to 7,326 (mean ± SD: 3087.0 ± 954.5, cumulative total citations = 308,701; Table 1). The 23 highest-ranked (Rank B) articles on the list received one-third of the total citations of these 100 articles, whereas the next and last one-third citations were contributed by the next 34 and 43 articles respectively, indicating that the distribution did not fulfill Bradford's law (Table 1). The normalized citation counts ranged from 0.007 to 0.163 (mean ± SD: 0.054 ± 0.028, Table 1). In terms of the total citation count (Rank B), the top-ranked article was published by Kurtzke (1983), which reported a scale evaluating neurologic impairment in patients with multiple sclerosis. With regard to the normalized citation count (Rank A), the top-ranked article was published by Nowak et al. (1984), which described the role of magnesium ions in modulating the response of glutamate-activated channels in neurons. The 100 most-cited articles were mainly research articles (*n* = 67), followed by reviews (*n* = 29), editorials (*n* = 2), letters (*n* = 1) and notes (*n* = 1).

**Table 1:** List of 100 most-cited neuroscience articles ranked according to their citation counts.

**Rank A^a^**	**Rank B^b^**	**Year**	**Authors and title**	**Journal**	**Normalized citation count^c^**	**10-year citation count^d^**	**Total citation count**	**Topic^e^**
1	34	1984	Nowak, L., Bregestovski, P., Ascher, P., Herbet, A., Prochiantz, A. Magnesium gates glutamate-activated channels in mouse central neurons	Nature	0.163	1,439	3,240	6
2	19	1988	Choi, D. W. Glutamate neurotoxicity and diseases of the nervous-system	Neuron	0.145	1,764	3,927	6
3	57	1987	Johnson, J. W., Ascher, P. Glycine potentiates the NMDA response in cultured mouse-brain neurons	Nature	0.135	1,511	2,625	6
4	62	1982	Whitehouse, P. J., Price, D. L., Struble, R. G., Clark, A. W., Coyle, J. T., Delong, M. R. Alzheimer's disease and senile dementia - loss of neurons in the basal forebrain	Science	0.121	897	2,568	1
5	70	1976	Davies, P., Maloney, A. J. F. Selective loss of central cholinergic neurons in Alzheimer's disease	Lancet	0.105	403	2,431	1
6	78	1990	Sokoloff, P., Giros, B., Martres, M. P., Bouthenet, M. L., Schwartz, J. C. Molecular-cloning and characterization of a novel dopamine receptor (D3) as a target for neuroleptics	Nature	0.103	1,451	2,346	2
7	85	1991	Garthwaite, J. Glutamate, nitric-oxide and cell cell signaling in the nervous-system	Trends Neurosci	0.102	1,563	2,266	6
8	9	2000	Banchereau, J., Briere, F., Caux, C., Davoust, J., Lebecque, S., Liu, Y. T., Pulendran, B., Palucka, K. Immunobiology of dendritic cells	Annu Rev Immunol	0.099	2,865	4,392	6
9	61	1991	Oppenheim, R. W. Cell-death during development of the nervous-system	Annu Rev Neurosci	0.098	1,501	2,579	6
10	96	1992	Nakanishi, S. Molecular diversity of glutamate receptors and implications for brain-function	Science	0.093	1,523	2,186	6
11	51	1993	Saunders, A. M., Strittmatter, W. J., Schmechel, D., Georgehyslop, P. H. S., Pericakvance, M. A., Joo, S. H., Rosi, B. L., Gusella, J. F., Crappermaclachlan, D. R., Alberts, M. J., Hulette, C., Crain, B., Goldgaber, D., Roses, A. D. Association of apolipoprotein-e allele epsilon-4 with late-onset familial and sporadic Alzheimer's disease	Neurology	0.084	1,486	2,719	1
12	14	1972	Racine, R. J. Modification of seizure activity by electrical stimulation. Part 2. Motor seizure	Electroencephalogr Clin Neurophysiol	0.084	196	4,227	1
13	16	2007	Louis, D. N., Ohgaki, H., Wiestler, O. D., Cavenee, W. K., Burger, P. C., Jouvet, A., Scheithauer, B. W., Kleihues, P. The 2007 WHO classification of tumours of the central nervous system	Acta Neuropathol	0.083	3,972	3,998	1
14	11	1997	Polymeropoulos, M. H., Lavedan, C., Leroy, E., Ide, S. E., Dehejia, A., Dutra, A., Pike, B., Root, H., **Rubenstein, J**., Boyer, R., Stenroos, E. S., Chandrasekharappa, S., Athanassiadou, A., Papapetropoulos, T., Johnson, W. G., Lazzarini, A. M., Duvoisin, R. C., DiIorio, G., Golbe, L. I., Nussbaum, R. L. Mutation in the alpha-synuclein gene identified in families with Parkinson's disease	Science	0.082	1,956	4,321	1
15	47	1993	Coyle, J. T., Puttfarcken, P. Oxidative stress, glutamate, and neurodegenerative disorders	Science	0.077	1,366	2,866	6
16	17	2001	McDonald, W. I., Compston, A., Edan, G., Goodkin, D., Hartung, H. P., Lublin, F. D., McFarland, H. F., Paty, D. W., Polman, C. H., Reingold, S. C., Sandberg-Wollheim, M., Sibley, W., Thompson, A. J., van den Noort, S., Weinshenker, B. Y., Wolinsky, J. S. Recommended diagnostic criteria for multiple sclerosis: Guidelines from the International Panel on the Diagnosis of Multiple Sclerosis	Ann Neurol	0.076	2,326	3,993	1
17	53	1992	Kwong, K. K., Belliveau, J. W., Chesler, D. A., Goldberg, I. E., Weisskoff, R. M., Poncelet, B. P., Kennedy, D. N., Hoppel, B. E., Cohen, M. S., Turner, R., Cheng, H. M., Brady, T. J., Rosen, B. R. Dynamic magnetic-resonance-imaging of human brain activity during primary sensory stimulation	Pro Natl Acad Sci U S A	0.074	1,220	2,702	5
18	25	1984	Hopfield, J. J. Neurons with graded response have collective computational properties like those of 2-state neurons	Pro Natl Acad Sci U S A	0.073	644	3,496	6
19	2	1991	**Braak, H.**, Braak, E. Neuropathological staging of Alzheimer-related changes	Acta Neuropathol	0.071	1,086	6,836	1
20	21	1998	Prusiner, S. B. Prions	Pro Natl Acad Sci U S A	0.068	1,745	3,837	1
21	27	2000	Schwartz, M. W., Woods, S. C., Porte, D., Seeley, R. J., Baskin, D. G. Central nervous system control of food intake	Nature	0.068	1,958	3,410	4
22	35	1991	Mirra, S. S., Heyman, A., McKeel, D., Sumi, S. M., Crain, B. J., Brownlee, L. M., Vogel, F. S., Hughes, J. P., Vanbelle, G., Berg, L. The Consortium to Establish a Registry for Alzheimer's Disease (CERAD). Part 2. Standardization of the neuropathologic assessment of alzheimers-disease	Neurology	0.067	1,029	3,226	1
23	5	2001	Miller, E. K., **Cohen, J. D**. An integrative theory of prefrontal cortex function	Annu Rev Neurosci	0.066	2,014	4,754	2
24	56	2009	**Bullmore, E. T., Sporns, O**. Complex brain networks: graph theoretical analysis of structural and functional systems	Nat Rev Neurosci	0.065	2,652	2,677	3
25	81	1982	Pulsinelli, W. A., Brierley, J. B., Plum, F. Temporal profile of neuronal damage in a model of transient forebrain ischemia	Ann Neurol	0.064	478	2,326	6
26	15	1993	Robinson, T. E., Berridge, K. C. The neural basis of drug craving - an incentive-sensitization theory of addiction	Brain Res Rev	0.063	1,113	4,016	2
27	36	2004	Rizzolatti, G., Craighero, L. The mirror-neuron system	Annu Rev Neurosci	0.063	2,315	3,150	6
28	7	2002	**Corbetta, M.**, Shulman, G. L. Control of goal-directed and stimulus-driven attention in the brain	Nat Rev Neurosci	0.062	2,028	4,515	4
29	18	2000	**LeDoux, J. E**. Emotion circuits in the brain	Annu Rev Neurosci	0.062	1,797	3,977	2
30	12	1995	Bell, A. J., Sejnowski, T. J. An information maximization approach to blind separation and blind deconvolution	Neural Comput	0.062	1,276	4,318	5
31	89	1993	Lin, L. F. H., Doherty, D. H., Lile, J. D., Bektesh, S., Collins, F. GDNF - a glial-cell line derived neurotrophic factor for midbrain dopaminergic-neurons	Science	0.061	1,085	2,256	2
32	88	1996	TessierLavigne, M., Goodman, C. S. The molecular biology of axon guidance	Science	0.060	1,340	2,258	6
33	58	1996	McKeith, I. G., **Galasko, D.**, Kosaka, K., Perry, E. K., **Dickson, D. W.**, Hansen, L. A., Salmon, D. P., Lowe, J., Mirra, S. S., Byrne, E. J., Lennox, G., Quinn, N. P., Edwardson, J. A., Ince, P. G., Bergeron, C., Burns, A., **Miller, B. L.**, Lovestone, S., Collerton, D., Jansen, E. N. H., Ballard, C., deVos, R. A. I., Wilcock, G. K., Jellinger, K. A., Perry, R. H. Consensus guidelines for the clinical and pathologic diagnosis of dementia with Lewy bodies (DLB): Report of the consortium on DLB international workshop	Neurology	0.060	1,332	2,614	1
34	41	2001	Logothetis, N. K., Pauls, J., Augath, M., Trinath, T., Oeltermann, A. Neurophysiological investigation of the basis of the fMRI signal	Nature	0.059	1,802	3,054	5
35	72^*b*^	1982	Kirino, T. Delayed neuronal death in the gerbil hippocampus following ischemia	Brain Res	0.059	436	2,400	3
36	45	2000	**Gage, F. H**. Mammalian neural stem cells	Science	0.058	1,679	2,891	6
37	48	2005	Polman, C. H., Reingold, S. C., Edan, G., Filippi, M., Hartung, H. P., **Kappos, L.**, Lublin, F. D., Metz, L. M., McFarland, H. F., O'Connor, P. W., Sandberg-Wollheim, M., Thompson, A. J., **Weinshenker, B. G.**, Wolinsky, J. S. Diagnostic criteria for multiple sclerosis: 2005 Revisions to the “McDonald Criteria”	Ann Neurol	0.057	2,304	2,842	1
38	10	2000	Ashburner, J., **Friston, K. J**. Voxel-based morphometry - The methods	NeuroImage	0.057	1,638	4,327	5
39	13	1999	**Petersen, R. C.**, Smith, G. E., Waring, S. C., Ivnik, R. J., Tangalos, E. G., Kokmen, E. Mild cognitive impairment - Clinical characterization and outcome	Arch Neurol	0.056	1,537	4,234	1
40	46	1998	Sakurai, T., Amemiya, A., Ishii, M., Matsuzaki, I., Chemelli, R. M., Tanaka, H., Williams, S. C., Richardson, J. A., Kozlowski, G. P., Wilson, S., Arch, J. R. S., Buckingham, R. E., Haynes, A. C., Carr, S. A., Annan, R. S., McNulty, D. E., Liu, W. S., Terrett, J. A., Elshourbagy, N. A., Bergsma, D. J., Yanagisawa, M. Orexins and orexin receptors: A family of hypothalamic neuropeptides and G protein-coupled receptors that regulate feeding behavior	Cell	0.055	1,412	2,879	6
41	40	2005	**Fox, M. D., Snyder, A. Z., Vincent, J. L., Corbetta, M., Van Essen, D. C., Raichle, M. E**. The human brain is intrinsically organized into dynamic, anticorrelated functional networks	Pro Natl Acad Sci U S A	0.054	2,151	3,078	3
42	42	2005	Ashburner, J., **Friston, K. J**. Unified segmentation	NeuroImage	0.053	2,131	3,051	5
43	31	1998	Eriksson, P. S., Perfilieva, E., Bjork-Eriksson, T., Alborn, A. M., Nordborg, C., Peterson, D. A., **Gage, F. H**. Neurogenesis in the adult human hippocampus	Nat Med	0.053	1,360	3,306	6
44	3	2002	Tzourio-Mazoyer, N., Landeau, B., Papathanassiou, D., Crivello, F., Etard, O., Delcroix, N., Mazoyer, B., Joliot, M. Automated anatomical labeling of activations in SPM using a macroscopic anatomical parcellation of the MNI MRI single-subject brain	NeuroImage	0.051	1,672	5,297	5
45	33	2003	**Braak, H., Del Tredici, K.**, Rub, U., de Vos, R. A. I., Steur, ENHJ, Braak, E. Staging of brain pathology related to sporadic Parkinson's disease	Neurobiol Aging	0.051	1,775	3,251	1
46	49	2002	Genovese, C. R., Lazar, N. A., Nichols, T. Thresholding of statistical maps in functional neuroimaging using the false discovery rate	NeuroImage	0.051	1,654	2,837	5
47	83	1994	Monyer, H., Burnashev, N., Laurie, D. J., Sakmann, B., Seeburg, P. H. Developmental and regional expression in the rat-brain and functional-properties of 4 NMDA receptors	Neuron	0.051	970	2,287	6
48	59	2002	**Walsh, D. M.**, Klyubin, I., Fadeeva, J. V., Cullen, W. K., Anwyl, R., Wolfe, M. S., Rowan, M. J., **Selkoe, D. J**. Naturally secreted oligomers of amyloid beta protein potently inhibit hippocampal long-term potentiation in vivo	Nature	0.051	1,652	2,611	1
49	72^*b*^	2007	**Fox, M. D., Raichle, M. E**. Spontaneous fluctuations in brain activity observed with functional magnetic resonance imaging	Nat Rev Neurosci	0.050	278	2,400	1
50	37	2000	Bush, G., Luu, P., Posner, M. I. Cognitive and emotional influences in anterior cingulate cortex	Trends Cogn Sci	0.050	1,438	3,138	4
51	38	1999	Pruessmann, K. P., Weiger, M., Scheidegger, M. B., Boesiger, P. SENSE: Sensitivity encoding for fast MRI	Magn Reson Med	0.049	1,326	3,130	5
52	95	2000	Cabeza, R., Nyberg, L. Imaging cognition II: An empirical review of 275 PET and fMRI studies	J Cogn Neurosci	0.048	1,393	2,190	4
53	50	1996	Kreutzberg, G. W. Microglia: A sensor for pathological events in the CNS	Trends Neurosci	0.048	1,057	2,750	6
54	24	2004	**Smith, S. M., Jenkinson, M., Woolrich, M. W., Beckmann, C. F., Behrens, T. E. J., Johansen-Berg, H.**, Bannister, P. R., De Luca, M., Drobnjak, I., Flitney, D. E., Niazy, R. K., Saunders, J., Vickers, J., Zhang, Y. Y., De Stefano, N., Brady, J. M., **Matthews, P. M**. Advances in functional and structural MR image analysis and implementation as FSL	NeuroImage	0.047	1,752	3,618	5
55	63	1994	Gurney, M. E., Pu, H. F., Chiu, A. Y., Dalcanto, M. C., Polchow, C. Y., Alexander, D. D., Caliendo, J., Hentati, A., Kwon, Y. W., Deng, H. X., Chen, W. J., Zhai, P., Sufit, R. L., Siddique, T. Motor-neuron degeneration in mice that express a human cu,zn superoxide-dismutase mutation	Science	0.047	892	2,551	1
56	80	2003	Santarelli, L., Saxe, M., Gross, C., Surget, A., Battaglia, F., Dulawa, S., Weisstaub, N., Lee, J., **Duman, R.**, Arancio, O., Belzung, C., **Hen, R**. Requirement of hippocampal neurogenesis for the behavioral effects of antidepressants	Science	0.046	1,605	2,332	2
57	92	2007	Haass, C., **Selkoe, D. J**. Soluble protein oligomers in neurodegeneration: lessons from the Alzheimer's amyloid beta-peptide	Nat Rev Mol Cell Bio	0.046	2,193	2,213	1
58	22	1997	Kanwisher, N., McDermott, J., Chun, M. M. The fusiform face area: A module in human extrastriate cortex specialized for face perception	J Neurosci	0.046	1,097	3,756	4
59	91	2007	Ashburner, J. A fast diffeomorphic image registration algorithm	NeuroImage	0.046	2,185	2,226	5
60	4	2001	**Raichle, M. E.**, MacLeod, A. M., **Snyder, A. Z.**, Powers, W. J., Gusnard, D. A., Shulman, G. L. A default mode of brain function	Pro Natl Acad Sci U S A	0.046	1,395	4,935	3
61	77	2006	**Smith, S. M., Jenkinson, M., Johansen-Berg, H.**, Rueckert, D., Nichols, T. E., Mackay, C. E., Watkins, K. E., Ciccarelli, O., Cader, M. Z., **Matthews, P. M., Behrens, T. E. J**. Tract-based spatial statistics: Voxelwise analysis of multi-subject diffusion data	NeuroImage	0.045	1,955	2,376	5
62	55	2001	Good, C. D., Johnsrude, I. S., Ashburner, J., Henson, R. N. A., **Friston, K. J.**, Frackowiak, R. S. J. A voxel-based morphometric study of ageing in 465 normal adult human brains	NeuroImage	0.045	1,366	2,678	5
63	66	2001	Danbolt, N. C. Glutamate uptake	Prog Neurobiol	0.045	1,366	2,496	6
64	64	1975	Hachinski, V. C., Iliff, L. D., Zilhka, E., Duboulay, G. H., McAllister, V. L., Marshall, J., Russell, R. W. R., Symon, L. Cerebral blood-flow in dementia	Arch Neurol	0.044	156	2,547	1
65	75	1999	Doetsch, F., Caille, I., Lim, D. A., Garcia-Verdugo, J. M., **Alvarez-Buylla, A**. Subventricular zone astrocytes are neural stem cells in the adult mammalian brain	Cell	0.044	1,206	2,387	6
66	100	2006	Neumann, M., Sampathu, D. M., Kwong, L. K., Truax, A. C., Micsenyi, M. C., Chou, T. T., Bruce, J., Schuck, T., **Grossman, M.**, Clark, C. M., McCluskey, L. F., **Miller, B. L., Masliah, E., Mackenzie, I. R., Feldman, H.**, Feiden, W., Kretzschmar, H. A., **Trojanowski, J. Q., Lee, V. M. Y**. Ubiquitinated TDP-43 in frontotemporal lobar degeneration and amyotrophic lateral sclerosis	Science	0.043	1,877	2,138	1
67	67	2004	**Petersen, R. C**. Mild cognitive impairment as a diagnostic entity	J Intern Med	0.042	1,541	2,495	1
68	71	2001	**Petersen, R. C.**, Doody, R., Kurz, A., Mohs, R. C., **Morris, J. C.**, Rabins, P. V., Ritchie, K., **Rossor, M.**, Thal, L., **Winblad, B**. Current concepts in mild cognitive impairment	Arch Neurol	0.041	1,259	2,412	1
69	6	1992	Hughes, A. J., Daniel, S. E., Kilford, L., **Lees, A. J**. Accuracy of clinical-diagnosis of idiopathic Parkinson's disease: a clinicopathological study of 100 cases	J Neurol Neurosurg Psychiatry	0.041	674	4,533	1
70	76	2000	Akiyama, H., Barger, S., Barnum, S., Bradt, B., Bauer, J., Cole, G. M., Cooper, N. R., Eikelenboom, P., Emmerling, M., Fiebich, B. L., Finch, C. E., Frautschy, S., Griffin, W. S. T., Hampel, H., Hull, M., Landreth, G., Lue, L. F., Mrak, R., **Mackenzie, I. R.**, McGeer, P. L., O'Banion, M. K., Pachter, J., Pasinetti, G., Plata-Salaman, C., Rogers, J., Rydel, R., Shen, Y., Streit, W., Strohmeyer, R., Tooyoma, I., Van Muiswinkel, F. L., Veerhuis, R., Walker, D., Webster, S., Wegrzyniak, B., Wenk, G., Wyss-Coray, T. Inflammation and Alzheimer's disease	Neurobiol Aging	0.041	1,184	2,378	1
71	20	2002	**Smith, S. M**. Fast robust automated brain extraction	Hum Brain Mapp	0.041	1,320	3,867	5
72	93	2005	McKeith, I. G., **Dickson, D. W.**, Lowe, J., Emre, M., O'Brien, J. T., **Feldman, H.**, Cummings, J., Duda, J. E., Lippa, C., Perry, E. K., **Aarsland, D.**, Arai, H., Ballard, C. G., **Boeve, B.**, Burn, D. J., Costa, D., Del Ser, T., Dubois, B., **Galasko, D.**, Gauthier, S., Goetz, C. G., Gomez-Tortosa, E., Halliday, G., Hansen, L. A., **Hardy, J.**, Iwatsubo, T., Kalaria, R. N., Kaufer, D., Kenny, R. A., Korczyn, A., Kosaka, K., **Lee, V. M. Y.**, Lees, A., Litvan, I., Londos, E., Lopez, O. L., Minoshima, S., Mizuno, Y., Molina, J. A., Mukaetova-Ladinska, E. B., Pasquier, F., Perry, R. H., Schulz, J. B., **Trojanowski, J. Q.**, Yamada, M. Diagnosis and management of dementia with Lewy bodies - Third report of the DLB consortium	Neurology	0.040	1,616	2,201	1
73	54	2002	Wolpaw, J. R., Birbaumer, N., McFarland, D. J., Pfurtscheller, G., Vaughan, T. M. Brain-computer interfaces for communication and control	Clin Neurophysiol	0.039	1,271	2,686	5
74	28	1992	Reynolds, B. A.,Weiss, S. Generation of neurons and astrocytes from isolated cells of the adult mammalian central-nervous-system	Science	0.039	642	3,389	6
75	52	2003	Maldjian, J. A., Laurienti, P. J., Kraft, R. A., Burdette, J. H. An automated method for neuroanatomic and cytoarchitectonic atlas-based interrogation of fMRI data sets	NeuroImage	0.039	1,340	2,708	5
76	1	1983	Kurtzke, J. F. Rating neurologic impairment in multiple-sclerosis: an Expanded Disability Status Scale (EDSS)	Neurology	0.038	310	7,326	1
77	98	1991	Terry, R. D., **Masliah, E.**, Salmon, D. P., Butters, N., Deteresa, R., Hill, R., Hansen, L. A., Katzman, R. Physical basis of cognitive alterations in Alzheimer's disease: synapse loss is the major correlate of cognitive impairment	Ann Neurol	0.037	574	2,182	1
78	43	1998	Neary, D., Snowden, J. S., Gustafson, L., Passant, U., Stuss, D., Black, S., Freedman, M., Kertesz, A., Robert, P. H., Albert, M., Boone, K., **Miller, B. L.**, Cummings, J., Benson, D. F. Frontotemporal lobar degeneration - A consensus on clinical diagnostic criteria	Neurology	0.037	947	3,042	1
79	97	1996	Lublin, F. D., Reingold, S. C. Defining the clinical course of multiple sclerosis: Results of an international survey	Neurology	0.034	753	2,183	1
80	30	1997	Schultz, W., Dayan, P., Montague, P. R. A neural substrate of prediction and reward	Science	0.034	806	3,367	2
81	32	2002	**Jenkinson, M.**, Bannister, P., Brady, M., Smith, S. Improved optimization for the robust and accurate linear registration and motion correction of brain images	NeuroImage	0.033	1,079	3,274	5
82	87	1998	Lambert, M. P., Barlow, A. K., Chromy, B. A., Edwards, C., Freed, R., Liosatos, M., Morgan, T. E., Rozovsky, I., Trommer, B., Viola, K. L., Wals, P., Zhang, C., Finch, C. E., Krafft, G. A., Klein, W. L. Diffusible, nonfibrillar ligands derived from A beta(1-42) are potent central nervous system neurotoxins	Pro Natl Acad Sci U S A	0.032	827	2,263	1
83	8	1996	**Cox, R. W**. AFNI: Software for analysis and visualization of functional magnetic resonance neuroimages	Comput Biomed Res	0.032	708	4,406	5
84	99	1998	Schultz, W. Predictive reward signal of dopamine neurons	J Neurophysiol	0.031	798	2,159	2
85	60	2001	**Jenkinson, M.**, Smith, S. A global optimisation method for robust affine registration of brain images	Med Image Anal	0.026	804	2,594	5
86	82	2002	Nichols, T. E., Holmes, A. P. Nonparametric permutation tests for functional neuroimaging: A primer with examples	Hum Brain Mapp	0.026	834	2,296	5
87	26	1994	Cummings, J. L., Mega, M., Gray, K., Rosenbergthompson, S., Carusi, D. A., Gornbein, J. The neuropsychiatric inventory - comprehensive assessment of psychopathology in dementia	Neurology	0.025	479	3,453	1
88	86	2001	Zhang, Y. Y., Brady, M., Smith, S. Segmentation of brain MR images through a hidden Markov random field model and the expectation-maximization algorithm	IEEE Trans Med Imaging	0.025	765	2,265	5
89	94	1999	Klimesch, W. EEG alpha and theta oscillations reflect cognitive and memory performance: a review and analysis	Brain Res Rev	0.024	659	2,196	1
90	69	1999	Pfurtscheller, G., da Silva, F. H. L. Event-related EEG/MEG synchronization and desynchronization: basic principles	Clin Neurophysiol	0.023	639	2,448	5
91	68	2002	**Fischl, B., Salat, D. H.**, Busa, E., Albert, M., Dieterich, M., Haselgrove, C., van der Kouwe, A., Killiany, R., Kennedy, D., Klaveness, S., Montillo, A., **Makris, N.**, Rosen, B., **Dale, A. M**. Whole brain segmentation: Automated labeling of neuroanatomical structures in the human brain	Neuron	0.021	699	2,491	5
92	90	1996	Gallese, V., Fadiga, L., Fogassi, L., Rizzolatti, G. Action recognition in the premotor cortex	Brain	0.020	447	2,236	4
93	44	1994	Chaplan, S. R., Bach, F. W., Pogrel, J. W., Chung, J. M., Yaksh, T. L. Quantitative assessment of tactile allodynia in the rat paw	J Neurosci Methods	0.019	366	2,948	6
94	79	1995	Forman, S. D., Cohen, J. D., Fitzgerald, M., Eddy, W. F., **Mintun, M. A.**, Noll, D. C. Improved assessment of significant activation in functional magnetic-resonance-imaging (fMRI) - use of a cluster-size threshold	Magn Reson Med	0.019	392	2,342	5
95	84	1998	Sled, J. G., Zijdenbos, A. P., **Evans, A. C**. A nonparametric method for automatic correction of intensity nonuniformity in MRI data	IEEE Trans Med Imaging	0.018	461	2,273	5
96	72^b^	1992	**Hardy, J. A.**, Higgins, G. A. Alzheimer's disease: the amyloid cascade hypothesis	Science	0.017	2,382	2,400	6
97	23	1993	**Morris, J. C**. The Clinical Dementia Rating (CDR) - current version and scoring rules	Neurology	0.016	282	3,621	1
98	39	1999	**Dale, A. M., Fischl, B.**, Sereno, M. I. Cortical surface-based analysis - I. Segmentation and surface reconstruction	NeuroImage	0.014	381	3,099	5
99	65	1994	Bechara, A., Damasio, A. R., Damasio, H., Anderson, S. W. Insensitivity to future consequences following damage to human prefrontal cortex	Cognition	0.014	265	2,533	2
100	29	1995	**Biswal, B.**, Yetkin, F. Z., Haughton, V. M., Hyde, J. S. Functional connectivity in the motor cortex of resting human brain using echo-planar MRI	Magn Reson Med	0.007	152	3,377	3

*Authors in bold indicated they were Highly Cited Researchers 2016 announced by Clarivate Analytics*.

a *Rank A ranked the articles according to their normalized citation counts*.

b *Rank B ranked the articles according to their total citation counts. Three articles have same rank as they have same number of total citations*.

c *Normalized citation count of a paper = its average annual citation count of 10 years since publication / average annual neuroscience publication count of that period*.

d *10-year citation count = citation count received during the first 10 years since publication*.

e *These 100 articles were evaluated and categorized into six topics – (1) neurological disorders, (2) prefrontal cortex and associated emotion/reward, (3) brain network, (4) brain mapping, (5) methodology, and (6) basic neuroscience*.

### Major contributing journals and periods

The 100 most-cited articles were published in 37 journals (Table 2). The journals that contributed five or more of the 100 most-cited articles included *Science, NeuroImage, Neurology, Nature* and *Proceedings of the National Academy of Sciences of the United States of America*. The journals that published the 100 most-cited neuroscience articles with the highest mean normalized citation counts were *Lancet, Annual Review of Immunology, Nature, Acta Neuropathologica* and *Trends in Neurosciences*. The 100 articles were published from 1972 to 2009, and the largest proportion of them was published during 1996–2000, followed by 2001–2005 and 1991–1995. Eighty-one of these 100 articles were published within these periods (Table 3).

**Table 2:** Journals in which the 100 most-cited neuroscience articles were published.

**Journal**	**Number of articles**	**Total citation count**	**Mean normalized citation count^a^ ± *SD***
Science	13	35,523	0.060 ± 0.028
NeuroImage	11	35,491	0.044 ± 0.012
Neurology	9	30,385	0.045 ± 0.022
Nature	6	17,286	0.096 ± 0.045
Proceedings of the National Academy of Sciences of the United States of America	6	20,311	0.058 ± 0.017
Annals of Neurology	4	11,343	0.059 ± 0.016
Annual Review of Neuroscience	4	14,460	0.072 ± 0.017
Archives of Neurology	3	9,193	0.047 ± 0.008
Brain Research^b^	3	8,612	0.049 ± 0.021
Magnetic Resonance in Medicine	3	8,849	0.025 ± 0.021
Nature Reviews Neuroscience	3	9,592	0.059 ± 0.008
Neuron	3	8,705	0.072 ± 0.064
Acta Neuropathologica	2	10,834	0.077 ± 0.009
Cell	2	5,266	0.050 ± 0.008
Clinical Neurophysiology	2	5,134	0.031 ± 0.011
Human Brain Mapping	2	6,163	0.033 ± 0.011
IEEE Transactions on Medical Imaging	2	4,538	0.021 ± 0.005
Neurobiology of Aging	2	5,629	0.046 ± 0.007
Trends in Neurosciences	2	5,016	0.075 ± 0.039
Annual Review of Immunology	1	4,392	0.099
Brain	1	2,236	0.020
Clinical Neurophysiology^c^	1	2,533	0.031
Cognition	1	4,406	0.014
Computers and Biomedical Research	1	4,227	0.032
Journal of Cognitive Neuroscience	1	2,190	0.048
Journal of Internal Medicine	1	2,495	0.042
Journal of Neurology Neurosurgery and Psychiatry	1	4,533	0.041
Journal of Neurophysiology	1	2,159	0.031
Journal of Neuroscience	1	3,756	0.046
Journal of Neuroscience Methods	1	2,948	0.019
Lancet	1	2,431	0.105
Medical Image Analysis	1	2,594	0.026
Nature Medicine	1	3,306	0.053
Nature Reviews Molecular Cell Biology	1	2,213	0.046
Neural Computation	1	4,318	0.062
Progress in Neurobiology	1	2,496	0.045
Trends in Cognitive Sciences	1	3,138	0.050

a *Normalized citation count of a paper = its average annual citation count of 10 years since publication/average annual neuroscience publication count of that period. Hence, mean normalized citation count of a journal = average of normalized citation counts of the top 100 most-cited neuroscience articles published in that journal*.

b *Two articles were published in Brain Research Reviews, merged into Brain Research since 2014*.

c *Clinical Neurophysiology was named Electroencephalography and Clinical Neurophysiology before 1999*.

**Table 3:** Distribution of the 100 most-cited neuroscience articles by publication date.

**Publication year**	**Number of articles**	**Mean normalized citation count^a^ ± *SD***
1971–1975	2	0.064 ± 0.028
1976–1980	1	0.105
1981–1985	6	0.086 ± 0.046
1986–1990	3	0.127 ± 0.022
1991–1995	23	0.051 ± 0.029
1996–2000	30	0.047 ± 0.019
2001–2005	28	0.046 ± 0.013
2006–2010	7	0.054 ± 0.015

a *Please refer to Table 2 for the definition of normalized citation count*.

### Major contributing authors

A total of 533 authors contributed to these 100 most-cited articles, and 55 of them were in the latest-available list of Highly Cited Researchers 2016. Ten authors contributed to three or more of the 100 most-cited articles (Table 4), led by Stephen M. Smith, John Ashburner, and Mark Jenkinson. All of them were still contributing to the academic publication in either 2016 or 2017. Among the 533 authors, we identified two Lasker Award winners, namely Prusiner, Stanley B., and DeLong, Mahlon R. We did not identify any Nobel Prize winner.

**Table 4:** Authors who contributed three or more of the 100 most-cited neuroscience articles.

**Author**	**Total articles^a^ (*n*)**	**Role of author in Total articles^b^**		**Mean normalized citation count ± *SD***
		**First and corresponding author**	**Co-author**	
**Smith, Stephen M**.	6	3	3	0.036 ± 0.009
Ashburner, John	4	3	1	0.050 ± 0.006
**Jenkinson, Mark**	4	2	2	0.038 ± 0.010
**Friston, Karl J**.	3		3	0.051 ± 0.006
Hansen, Lawrence A.	3		3	0.041 ± 0.004
Lublin, Fred D.	3	1	2	0.056 ± 0.021
**Miller, Bruce L**.	3		3	0.047 ± 0.012
**Petersen, Ronald C**.	3	3		0.046 ± 0.009
**Raichle, Marcus E**.	3	1	2	0.050 ± 0.004
Reingold, Stephen C.	3		3	0.056 ± 0.021

*Authors in bold indicated they were in the list of Highly Cited Researchers 2016 announced by Clarivate Analytics*.

a *Lublin and Reingold co-authored the same 3 articles. Miller and Raichle each contributed to one article that belonged to cluster 2 (articles published in higher impact factor journals). All other articles reported here belonged to cluster 1. Please refer to Figure 2 concerning definition of the clustering*.

b *None of these ten authors were first author but not corresponding or corresponding but not first author; all listed authors remain active, i.e., last indexed article published in 2016 or 2017*.

### Five topics formed a citation network

**Figure 1:**
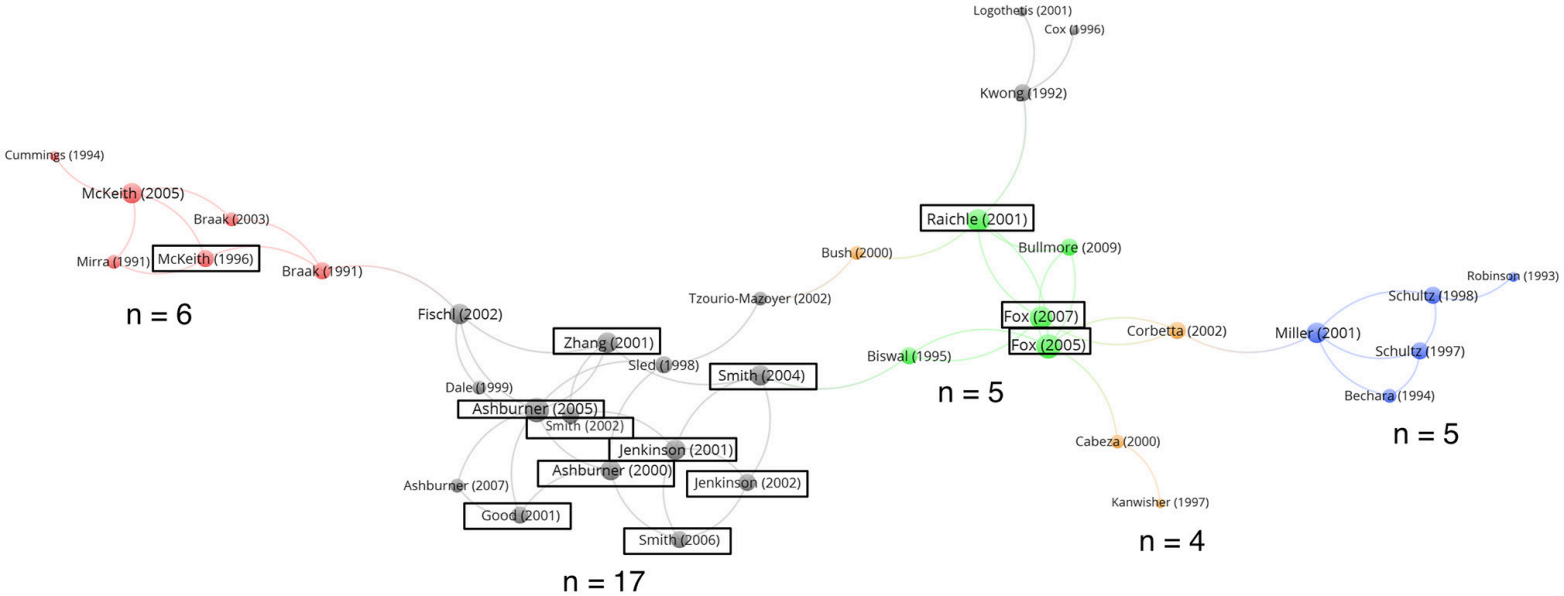
Citation network among the 100 most-cited neuroscience articles. Thirty-seven articles (first author and publication year) were interlinked with one another by 55 citations, thus forming the network. These articles could be classified into five topics: (1) neurological disorders (red, *n* = 6); (2) the prefrontal cortex and associated emotion/reward (blue, *n* = 5); (3) the brain network (green, *n* = 5); (4) brain mapping (orange, *n* = 4); and (5) the methodology (black, *n* = 17). The bubble size indicated the citation count received within this 37-article network. Articles surrounded by a black solid line had authorship from six Highly Cited Researchers who had at least three most-cited neuroscience articles, with more details as follows: Friston has authored “Ashburner (2000) and (2005)” and “Good (2001)” Jenkinson has authored “Jenkinson (2001) and (2002)” and “Smith (2004)” Miller has authored “McKeith (1996)” Petersen did not author articles in this network; Raichle has authored “Fox (2005) and (2007)” and Smith has authored “Jenkinson (2001) and (2002),” “Smith (2002), (2004) and (2006)” and “Zhang (2001).”

Thirty-seven of these 100 most-cited articles were interlinked with 55 citations among them, and the citation network is displayed in Figure 1. The articles forming the network could be classified into five topics, including four scientific topics: (1) neurological disorders, e.g., Alzheimer's disease, dementia, and Parkinson's disease (red); (2) the prefrontal cortex and associated emotion/ reward (blue); (3) the brain network, including the resting state network (green); and (4) brain mapping (orange). The remaining topic was methodology, e.g., the basis of functional magnetic resonance imaging signals, data analytic, computation, and processing methods (black). It seemed that brain connectivity papers (Figure 1, green) were at the center of the citation network surrounded by method (black dot) and brain mapping (orange) papers. Meanwhile the neurological disorders (red) and emotion/reward (blue) papers were at the periphery. It should be noted that the linkages among these five topics were relatively weak (only nine inter-topic citation links, Figure 1), and there was no cross-topic author. This indicated that the citations were mainly self-connecting within each topic, e.g., within the brain network and methodology groups, respectively. These five topics together accounted for 78 out of the 100 most-cited papers. Among these 78 papers, 52.6% (41/78) of them were not within the citation network.

We examined the remaining 63 articles not connected to the 37-article network by citations, and we discovered that 27 were related to neurological disorders, four were related to the prefrontal cortex and associated emotion/ reward, two were related to brain mapping and eight were related to methodology. Twenty-two articles were related to basic neuroscience. Overall, the articles of the five major topics (*n* = 78) contributed to 79.6% (neurological disorder: 34.3%, prefrontal cortex/emotion/reward: 9.0%, brain network: 5.3%, brain mapping: 6.3%, and methodology: 24.7%) of the 308,701 total citations that all of the 100 most-cited articles received. There was no cross-topic author among these included articles.

### Normalized citation influenced by years of publication

**Figure 2:**
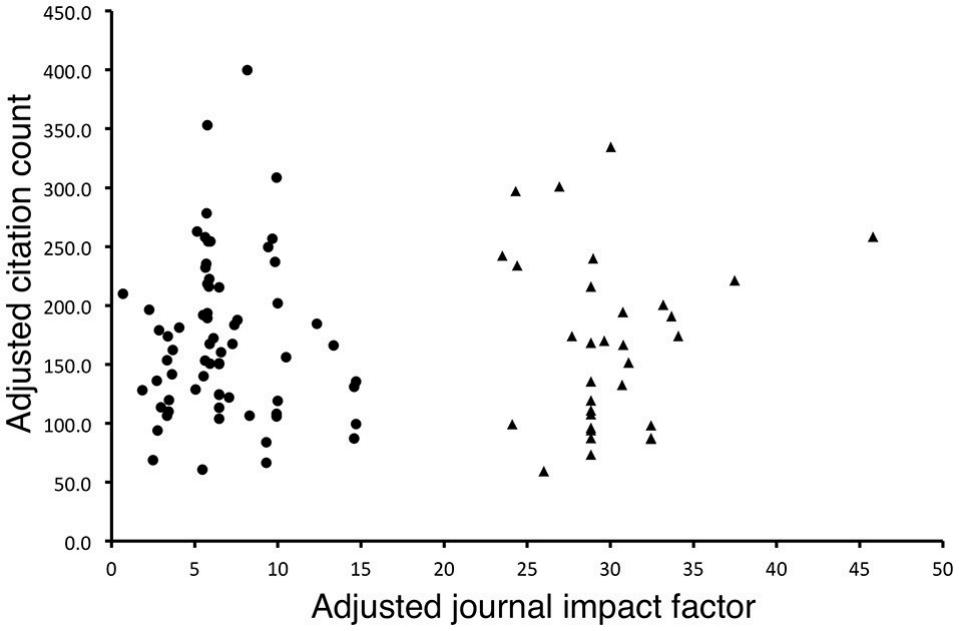
Plot of adjusted citation count against adjusted journal impact factor. There was no correlation between the two factors (*r*^2^ = –0.025, *p* = 0.804). The articles were divided into two clusters (cluster 1: circles and cluster 2: triangles), with cluster 2 (*n* = 32) consisting of articles published in journals with higher adjusted impact factors. There was no apparent difference in the adjusted citation count between the two clusters. In addition, there was no relationship between adjusted citation count and adjusted impact factor within each cluster (cluster 1: *r*^2^ = –0.033, *p* = 0.791; cluster 2: *r*^2^ = 0.079, *p* = 0.666). The adjusted citation count of an article = the total citation count/years of publication. The adjusted journal impact factor = the summation of annual impact factors since the publication of that particular article/years of publication.

We did not find a significant correlation between the adjusted journal impact factor and the adjusted citation count (*r*^2^ = −0.025, *p* = 0.804, Figure 2). Two-step clustering analysis found that the data points could be divided into two clusters with a silhouette value of 0.9, indicating that the clustering was distinct. The smaller cluster (cluster 2, triangles, Figure 2) consisted of 32 articles published in nine journals with relatively higher adjusted impact factors. Among these 32 articles, there were 22 Highly Cited Researchers 2016 authoring 14 papers. The six Highly Cited Researchers who authored at least three of the 100 most-cited neuroscience articles published only two articles in cluster 2. However, the normalized citation counts of articles in this cluster did not seem to differ much from those in the other cluster with a lower normalized impact factor. Also, within each cluster, there was no association detectable between the normalized citation count and the normalized impact factor.

**Figure 3:**
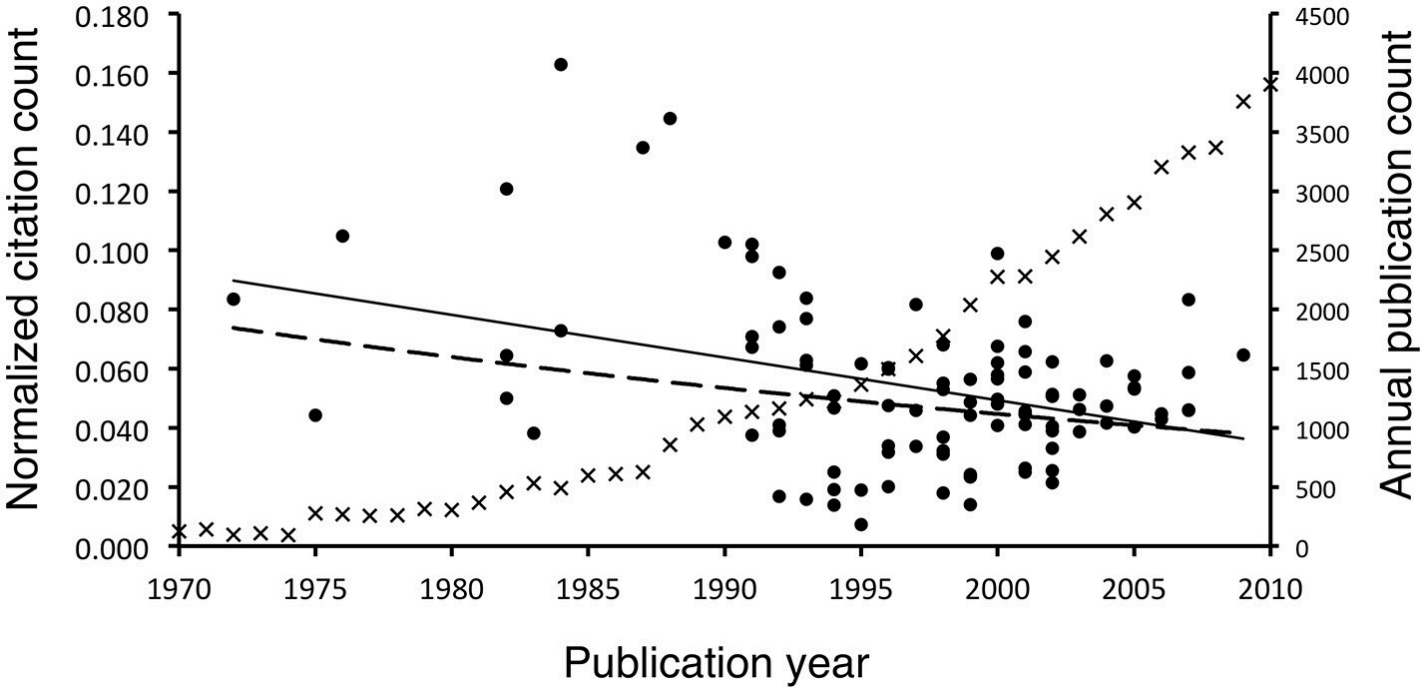
Plot of normalized citation count of the 100 most-cited neuroscience articles and annual neuroscience publication count against publication year. Each normalized citation count point was represented by a dot. It positively correlated (*r*^2^ = 0.373, *p* < 0.001) with years since publication (i.e., negatively correlated with publication year). The growth fitted a linear trend (dashed line, *r*^2^ = 0.14) better than an exponential trend (solid line, *r*^2^ = 0.06). For illustrative purpose, the annual count of neuroscience publications during the same period was plotted (each data was represented by a cross). It experienced an exponential growth (*y* = 3 × 10^−77^e^0.0919*x*^). Data were retrieved from searching “neuro^*^” in the PubMed database.

On the contrary, there was a positive correlation between years since publication of the article and the normalized citation count (*r*^2^ = 0.373, *p* < 0.001, Figure 3), meaning that newer publications had a lower normalized citation count. Such a correlation followed a best-fitting linear trend line (*r*^2^ = 0.14) more closely than an exponential one (*r*^2^ = 0.06), indicating that it does not fulfill Price's law. Conversely, the annual neuroscience publication counts during the same period experienced exponential growth (*r*^2^ = 0.96).

## Discussion

Similar to previous publications (Ponce and Lozano, 2010; Kim, E. S. et al., 2016; Kim, H. J. et al., 2016; Kreutzer et al., 2017), we evaluated the top 100 most-cited neuroscience articles by convenience. From the visualized citation network (Figure 1), it could be seen that the articles investigating the brain connectivity (green), particularly the resting state, were relatively published more recently (Raichle et al., 2001; Fox et al., 2005; Fox and Raichle, 2007; Bullmore and Sporns, 2009). This was consistent with the perspectives of Friston (2011) and Snyder and Raichle (2012) that the topic of brain connectivity, particularly during the resting state, has gained popularity and importance. Besides resting state, sensory studies have also employed connectivity analyses (Yeung et al., 2016). Such a research direction, together with basic cellular and molecular neuroscience, should provide extra insight into a better understanding of neurological disorders, which the neuroscience literature has already heavily emphasized. We observed that the topic of neurological disorders contributed six papers to the set of 37 interlinked papers, whereas there were 27 papers in this topic amongst the 63 non-interlinked papers. For the topics of methodology and brain connectivity, these numbers were 17/8 and 5/0 respectively. These five brain connectivity papers with high citation counts were reporting functional connectivity when the brain was at resting state; and thus they were highly relevant to each other and interlinked. Similarly, the majority of methodology papers with high citation counts were related to each other or followed by high-citation papers from other topics, and thus appeared in the interlinked network. However, perhaps the spectrum of neurological disorders was so diverse that a proportion of these highly cited papers were not related to each other and thus not appearing in the interlinked network.

The distribution of the citations that the 100 most-cited articles received did not follow Bradford's law. Instead, it was more evenly distributed among the articles. This implied that the 100 most-cited articles were receiving relatively balanced attention and citation counts instead of having a few articles among them continuing to dominate the neuroscience literature. The top two most-cited articles revealed by the current study were published decades ago, in 1983 and 1991, respectively, but did not rank very high under the normalized citation count (76 and 19, Table 1). On the other hand, the major contributing journals that published half of the 100 most-cited articles, perhaps except two, had long histories, namely *Science* (since 1880), *NeuroImage* (since 1993), *Neurology* (since 1951), *Nature* (since 1869), *Proceedings of the National Academy of Sciences of the United States of America* (since 1914) and *Annals of Neurology* (since 1977). This phenomenon was consistent with other subfield studies (Kim, E. S. et al., 2016; Kim, H. J. et al., 2016). From Figure 2 it can be seen that the articles were divided into two clusters by adjusted journal impact factor. The smaller cluster consisted of articles published in high-impact factor journals, such as *Cell, Lancet, Science*, the *Nature* group of journals, *Annual Review of Neuroscience*, and *Annual Review of Immunology*. However, there was no apparent difference in the adjusted citation counts of articles between the two clusters. Also, in each cluster there was no relationship between the adjusted citation count and the adjusted impact factor. This is consistent to previous reports on cardiovascular research that the journal impact factor is not representative of the citation count of its individual papers (Opthof, 1997; Opthof et al., 2004).

The results from the current study indicated that the periods that contributed to the largest proportion of the 100 articles were 1991–2005, which was later than the period (1976–1995) reported by a similar survey on neurosurgery (Ponce and Lozano, 2010) but was similar to the situations for neurointervention (Kim, E. S. et al., 2016) and neuroimaging (Kim, H. J. et al., 2016). In addition, we observed a significant correlation that the newer most-cited articles received a lower normalized citation count. Though the more recently published articles must have a higher annual citation count than their older counterparts to enter the list of 100 most-cited articles, including neuroscience articles (Lefaivre et al., 2011), the reduction in their normalized citation count could be explained by the exponential growth in neuroscience publications. The implication was that over the years the growth of neuroscience publication count was larger than the growth of citation count. These analyses should have essentially provided more balanced accounts for the citation analysis compared to those evaluations based on the total citation count only, as the articles with both high total and normalized citation counts should be the ones with up-to-date relevance for current and future researchers.

The averaged citation count of the 100 most-cited neuroscience articles was 3,087, which was higher than those reported in clinical/surgical subfields such as neurosurgery (452.6, Ponce and Lozano, 2010), neruointervention (363.5, Kim, E. S. et al., 2016), and neurorehabilitation (317.0, Kreutzer et al., 2017). This difference was consistent with general bibliometric findings that basic science researches often received more citations than did specific clinical researches (Van Eck et al., 2013; Yeung et al., 2017b). It should be noted that there was also opposite information which stated that clinical papers were more frequently cited than basic science papers, at least in the field of cardiovascular research. This implied that the balance between basic and clinical science may vary amongst fields (Opthof, 2011).

Review papers accounted for 29% of the top 100 most-cited articles in the current study, which was considerably higher than the proportion identified by subfield studies, such as 16% in neuroimaging (Kim, H. J. et al., 2016), 16% in neurorehabilitation (Kreutzer et al., 2017), 11% in neurosurgery (Ponce and Lozano, 2010), and even 0% in neurointervention (Kim, E. S. et al., 2016). This could imply that other subfields of neuroscience, such as the basic and cellular neurosciences, have published crucial reviews that received a lot of citations, such as the articles ranked 2, 8, and 27 (Rank A) on the top 100 list of the current study.

Approximately 10% of the authors (55 out of 533) who contributed to the 100 most-cited neuroscience articles were listed as Highly Cited Researchers 2016. Six of the ten authors who contributed to three or more most-cited neuroscience articles were Highly Cited Researchers 2016. The most-cited neuroscience articles of these six authors were mainly published in *NeuroImage* (33%). Only two of the most-cited neuroscience articles authored by them belonged to cluster 2 in Figure 2 (triangles, one in *Nature Reviews Neuroscience* and one in *Science*). Within the 37-article citation network, these six Highly Cited Researchers contributed to 13 articles (black solid line, Figure 1), accounting for nine of the 14 articles in the methodology group (black dot), three of the five articles in the brain network group (green) and one of the six articles in the neurological disorder group (red). These indicated they preferably focused on the methodology and brain network, and they published in *NeuroImage*.

There are several intrinsic limitations of using citation analysis to evaluate the academic importance of a specific article, author or journal. First, the interpretation of citation count may not be straightforward. For instance, it was impossible to identify inappropriate citations, citations for questioning or rebuttal instead of affirmation, and selective citation behaviors, such as preferably citing articles that were written in English, were available through open access and were published in prestigious journals (Garfield, 1979). In addition, through the “snowball effect,” people tend to cite publications that are already highly cited (Kuhn, 1962; Lefaivre et al., 2011). Second, the search was limited to the WoS database. It did not record citations by textbooks, and there might exist inaccurate or missing data especially regarding very ancient publications. There are other databases, such as Scopus and Google Scholar, but they are weaker at tracking older publications (Ponce and Lozano, 2010). In addition, from the citation network visualized in the current study there were five major topics and there were no cross-topic authors. This might imply that neuroscience research has different niches and it would be worthwhile for future bibliometric studies to further evaluate specific bodies of literature within neuroscience.

## Conclusions

We have identified the 100 most-cited articles in neuroscience to assess its driving force over time, and we have revealed that there were five major topics interlinked to one another, namely four scientific topics and one methodology. The list produced in the current study allows clinicians and scientists to quickly grasp important works and findings for their references.

## Author contributions

AY conceived the work, acquired and analyzed data, and drafted the work. TG and WL facilitated the acquisition of data and critically revised the work. All authors have approved the final content of the manuscript.

### Conflict of interest statement

The authors declare that the research was conducted in the absence of any commercial or financial relationships that could be construed as a potential conflict of interest.

## Footnotes

^1^http://hcr.stateofinnovation.com/.

^2^http://www.laskerfoundation.org/awards/.

^3^http://www.nobelprize.org/nobel_prizes/lists/all/).
